# Progranulin gene delivery reduces plaque burden and synaptic atrophy in a mouse model of Alzheimer's disease

**DOI:** 10.1371/journal.pone.0182896

**Published:** 2017-08-24

**Authors:** Jackalina M. Van Kampen, Denis G. Kay

**Affiliations:** 1 Neurodyn Inc., Charlottetown, PE, Canada; 2 Dept. Biomedical Science, University of Prince Edward Island, Charlottetown, PE, Canada; 3 Dept. Neuroscience, Mayo Clinic, Jacksonville, FL, United States of America; 4 Dept. Pathology and Microbiology, University of Prince Edward Island, Charlottetown, PE, Canada; Nathan S Kline Institute, UNITED STATES

## Abstract

Progranulin (PGRN) is a multifunctional protein that is widely expressed throughout the brain, where it has been shown to act as a critical regulator of CNS inflammation and also functions as an autocrine neuronal growth factor, important for long-term neuronal survival. PGRN has been shown to activate cell signaling pathways regulating excitoxicity, oxidative stress, and synaptogenesis, as well as amyloidogenesis. Together, these critical roles in the CNS suggest that PGRN has the potential to be an important therapeutic target for the treatment of various neurodegenerative disorders, particularly Alzheimer’s disease (AD). AD is the leading cause of dementia and is marked by the appearance of extracellular plaques consisting of aggregates of amyloid-β (Aβ), as well as neuroinflammation, oxidative stress, neuronal loss and synaptic atrophy. The ability of PGRN to target multiple key features of AD pathophysiology suggests that enhancing its expression may benefit this disease. Here, we describe the application of PGRN gene transfer using in vivo delivery of lentiviral expression vectors in a transgenic mouse model of AD. Viral vector delivery of the PGRN gene effectively enhanced PGRN expression in the hippocampus of Tg2576 mice. This elevated PGRN expression significantly reduced amyloid plaque burden in these mice, accompanied by reductions in markers of inflammation and synaptic atrophy. The overexpression of PGRN was also found to increase activity of neprilysin, a key amyloid beta degrading enzyme. PGRN regulation of neprilysin activity could play a major role in the observed alterations in plaque burden. Thus, PGRN may be an effective therapeutic target for the treatment of AD.

## Introduction

Progranulin (PGRN) is a 593 amino acid multifunctional secreted glycoprotein consisting of tandem repeats of granulin, a 12-cysteine module also called epithelin domain. In the CNS, PGRN is widely expressed and found primarily in neuronal and microglial populations [1, 2]. While relatively little is known about its function in the CNS, PGRN is thought to play a role in CNS inflammatory responses, consistent with its strong immunoreactivity in activated microglia[1]. This may explain reports of upregulated expression in numerous disease states involving microglial activation, including motor neuron disease, lysosomal storage disease, and Alzheimer’s disease[1] [3, 4]. Indeed, in mice lacking PGRN, inflammatory responses become dysregulated[5, 6]. It is also becoming increasingly apparent that PGRN may have neurotrophic properties, functioning as an autocrine neuronal growth factor, important for long-term neuronal survival [2, 7]. Indeed, the absence of PGRN has been found to render neuronal cells vulnerable to insult, both in vitro[8] and in vivo[5, 6]. Thus, PGRN functions in CNS diseases may be related to neuronal growth support and/or microglial immune responses and mutations in *PGRN* might influence susceptibility to a wide range of neurodegenerative diseases, including Alzehimer’s disease (AD).

An upsurge in PGRN research has occurred recently, owing to the association of *PGRN* mutations with neurodegenerative disease. The first link between PGRN and neurodegeneration came when *PGRN* mutations were first causally associated with ubiquitin-positive frontotemporal lobar degeneration linked to chromosome 17q21 (FTLDU-17) [1, 9]. Since then, more than 113 *PGRN* mutations have been identified. Despite the association with ubiquitin inclusions, the majority of these mutations are null mutations involving a simple loss of function rather than accumulation of mutant protein [1, 10]. Until recently, it has been primarily FTLDU associated with *PGRN* mutations. However, the clinical phenotype associated with these mutations is highly varied and includes features that resemble other neurodegenerative diseases, including AD [11, 12]. Alterations in PGRN levels have also been associated with Lewy body dementia[13] and AD[11, 14]. PGRN expression appears up-regulated in glial cells of both AD patients and transgenic mouse models, in association with plaques [1, 2, 15]. Collectively, recent findings suggest that PGRN may influence various aspects of AD pathology, including Aβ accumulation, neuroinflammation, and toxicity[16–18]. Thus, efforts to enhance PGRN expression in the CNS may have therapeutic potential for AD.

The Tg2576 mouse model of AD expresses the Swedish mutation of APP (APP_K67ON,M671L_) at high levels under the control of the hamster prion protein promoter. Levels of APP in the brains of these transgenic animals are more than 4 times higher than APP levels in control mice and Aβ levels are 5–14 times higher than Aβ levels in control mice[19]. These mice develop a progressive, age-related deposition in the form of amyloid plaques in the cortex and hippocampus. A rapid increase in insoluble Aβ occurs around 6 months of age and plaques begin to form around 8–12 months of age[20], resulting in the development of memory deficits[19, 21].

The present study was designed to determine the disease-modifying effects of enhanced PGRN expression on synaptic pathology and plaque growth in the Tg2576 mouse model of familial Alzheimer’s disease. By transducing hippocampal neurons using the lentiviral vector, ND-602, we show that ND-602 effectively increases PGRN expression in neurons of the hippocampus, reduces amyloid plaque burden, inflammation and synaptic atrophy. Alterations in activity of the rate-limiting Aβ degrading enzyme, neprilysin, are also reported, raising a possible mechanism of action.

## Methods

### Animals

Female Tg2576 mice (Charles River) were housed in a specific pathogen free, temperature-controlled environment with a 12 h light/dark cycle and 24 hour *ad libitum* access to standard chow and water. Animals were group housed in standard shoebox cages, with filter top lids and beta chip bedding. Mouse huts were placed in each cage for enrichment. Experimental groups were treated and assessed using a balanced design in all procedural aspects, including animal husbandry. All animal experimentation was conducted in accordance with the NIH and CCAC guidelines for the care and use of laboratory animals and were approved by the Mayo Foundation Institutional Animal Care and Use Committee (IACUC) and the University of Prince Edward Island institutional Animal Care Committee (ACC).

### Lentiviral construct

Progranulin- and GFP-expressing lentiviral vectors were generated under contract with Invitrogen Corporation (Carlsbad, CA), as previously described[22]. Briefly, entry clones were first generated containing the promotor of interest (CMV) and the gene of interest (m*GRN*). Mouse progranulin full-length cDNA plasmid, a RIKEN clone (clone# 2900053G23) contained the following amino acid sequence:

MWVLMSWLAFAAGLVAGTQCPDGQFCPVACCLDQGGANYSCCNP
LLDTWPRITSHHLDGSCQTHGHCPAGYSCLLTVSGTSSCCPFSKGVSCGDGYHCCPQG
FHCSADGKSCFQMSDNPLGAVQCPGSQFECPDSATCCIMVDGSWGCCPMPQASCCEDR
VHCCPHGASCDLVHTRCVSPTGTHTLLKKFPAQKTNRAVSLPFSVVCPDAKTQCPDDS
TCCELPTGKYGCCPMPNAICCSDHLHCCPQDTVCDLIQSKCLSKNYTTDLLTKLPGYP
VKEVKCDMEVSCPEGYTCCRLNTGAWGCCPFAKAVCCEDHIHCCPAGFQCHTEKGTCE
MGILQVPWMKKVIAPLRLPDPQILKSDTPCDDFTRCPTNNTCCKLNSGDWGCCPIPEA
VCCSDNQHCCPQGFTCLAQGYCQKGDTMVAGLEKIPARQTTPLQIGDIGCDQHTSCPV
GQTCCPSLKGSWACCQLPHAVCCEDRQHCCPAGYTCNVKARTCEKDVDFIQPPVLLTL
GPKVGNVECGEGHFCHDNQTCCKDSAGVWACCPYLKGVCCRDGRHCCPGGFHCSARGT

**Fig 1:**
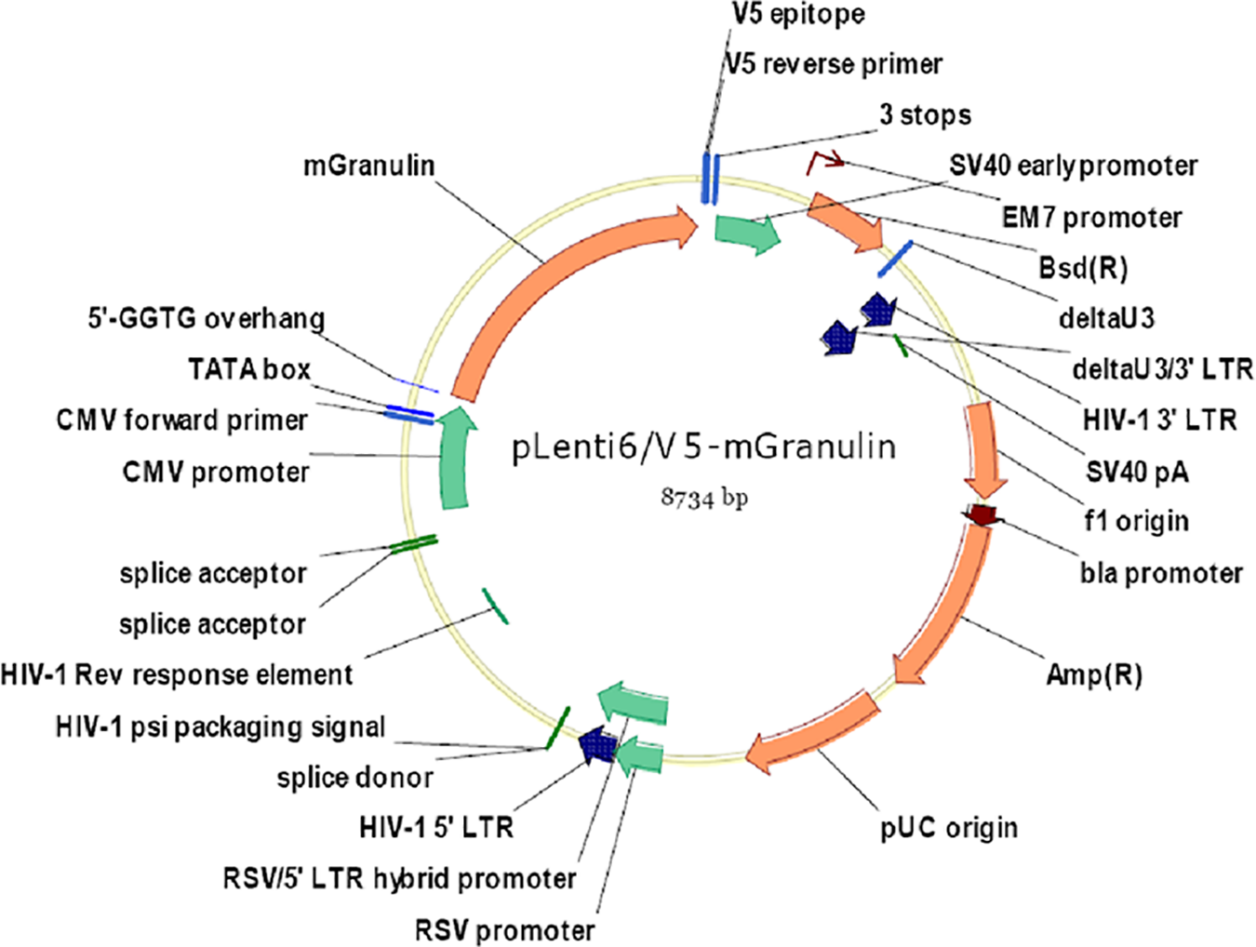
Lentiviral vector map. Representative map of the lentiviral contruct, ND-602. ND-602 is a viral vector construct designed for the targeted increase of mPGRN expression in the brain.

KCLRKKIPRWDMFLRDPVPRPLL. The CMV immediate early promoter allows high-level, constitutive expression of the gene of interest in mammalian cells. A Gateway LR Recombination was then performed to simultaneously transfer the two DNA fragments into the pLenti6/R4R2/V5-DEST vector using purified plasmid DNA from each entry clone to transform E. coli cells, creating an expression clone (Fig 1). The full pLenti6/V 5-mGranulin vector sequence can be found in the supporting file, S1 Appendix. The expression vectors were verified by transfecting the plasmid directly into a mammalian cell line, HEK 293 cells, and assaying for mPGRN or GFP expression. The 293FT cell line was then used to produce lentiviral stocks. The titer was determined to be 1X108 TU/mL, using a blasticidin resistance assay.

### Lentiviral delivery

At 8 months of age, when amyloid deposits first start to appear, animals received acute intrahippocampal infusion of either ND-602, or a GFP-expressing control vector. Thus, animals were anaesthetized using isoflurane (1%) and placed in a Kopf stereotaxic frame. The ND-602 was injected unilaterally the left hippocampus (A.P. -2.1, M.L. +1.50, D.V. -1.8) at a rate of 0.2 μl/minute via an infusion cannula connected by polyethylene tubing (50 PE) to a 50 μl Hamilton syringe driven by a Harvard pump. Following infusion, the viral vector was permitted to diffuse away from the cannula for two minutes before withdrawal. The antibiotic, sterile penicillin G Procaine (approximately 1,000 units i.m.) was administered along with the analgesic, Torbugesic (1 mg/kg, s.c.), for pain management. Animals were then placed on a heating pad until waking. Animals were monitored daily for signs of dehydration, infection, or excessive weight loss. However, no adverse indications were noted.

### Immunohistochemistry

Mice were sacrificed at 12 months of age by transcardial perfusion of PBS, the brains removed and post-fixed in 4% paraformaldehyde for immunohistochemical analysis. Symmetrical 30 μm-thick coronal sections were cut on a freezing microtome and stored in a Millonigs solution. Free-floating sections were pretreated with 70% formamide in Triton X-100/Tris-buffered saline [TBSt] at 37°C for 30 minutes and rinsed in TBSt. Sections were then incubated in 1% H_2_O_2_ in TBSt for 30 minutes, rinsed in TBSt, and incubated in blocking solution (5% goat serum/100mM lysine/0.3% TBSt) for 1 hour at room temperature, followed by incubation with the Aβ primary antibody (MM-27 33.1.1; 1:2000) overnight at room temperature. Sections were then incubated in a biotinylated secondary antibody followed by avidin-biotin-peroxidase complex using the Vectastain Elite kit. Sections were mounted on gelatin-coated slides and coverslipped with Entallen. For double immunostaining, sections were processed as described above using a secondary antibody conjugated to rhodamine. Sections were then rinsed in 1% Thioflavine-S (Sigma) for 20 minutes followed by 70% ethanol for 5 minutes, and several washes of distilled water. Other primary antibodies included GFP (Chemicon AB16901, polyclonal chicken anti-GFP, 1:600), PGRN (R&D Systems AF2557, polyclonal sheep anti-PGRN, 1:1000), synaptophysin (Abcam ab146921, polyclonal rabbit anti-synaptophysin, 1:1200), glial fibrillary acidic protein (GFAP) (Chemicon AB1540, polyclonal rabbit anti-GFAP, 1:1000), Iba1 (Abcam ab107159, polyclonal goat anti-Iba1, 1:2000), and neprilysin (Chemicon AB5458, polyclonal rabbit anti-neprilysin, 1:1000). For these, a heat-mediated antigen retrieval step, involving incubation in 2X SSC at 65°C for 20 minutes, was included. Sections were blocked with either goat or donkey serum (Millipore, 5%) for 1 hour prior to overnight incubation in primary antibody at 3°C. For fluorescent visualization, sections were incubated with the respective fluorescent secondary antibody conjugated to either Alexa 488, Alexa 594, or Alexa 350 (Molecular Probes, 1:500). Sections were also assayed for inflammation using fluorescein-conjugated isolectin B_4_ (ILB4) (Molecular Probes, 10μg/ml). Sections were mounted on unsubbed glass slides and coverslipped in Fluoromount. Fluorescence signals were detected with a Zeiss AxioObserver Z1 Imaging microscope equipped with an apotome system at excitation/emission wavelengths of 535/565 nm, 470/505 nm, and 585/615 nm.

### Neprilysin activity assay

Mice were perfused transcardially with PBS, after which the brains were quickly removed and the hippocampus and frontal cortex was dissected under a stereomicroscope. Each portion was homogenized with a motor-driven Teflon–glass homogenizer in five volumes (w/v) of ice-cold 10 mM Tris–HCl buffer (pH 8.0) containing 0.25 M sucrose, protease inhibitor cocktail (Complete™, EDTA-free, Roche Diagnostics, Indianapolis, IN) and 10 μM leupeptin. The homogenates were centrifuged at 9000×g and 4°C for 15 min and the supernatants were further centrifuged at 200 000×g and 4°C for 20 min using an Optima TL ultracentrifuge and a TLA100.4 rotor (Beckman, Palo Alto, CA). The pellets were solubilized in the Tris–HCl buffer containing 1% Triton X-100 (v/v) for 1 h on ice. The solubilized membranes were re-centrifuged at 200 000×g and 4°C for 20 min. The resultant clear supernatants were used as the membrane fraction. The standard assay mixture consisted of a 40 μg membrane fraction, 50 μM Z-Ala–Ala–Leu–p-nitroanilide (R&D Systems) as a substrate, and 50 mM MES buffer (pH 6.5) in a total volume of 100 μl. The reaction was initiated by addition of substrate to the assay mixture and performed at 37°C for 30 min. The neprilysin activity was determined by monitoring the absorbance of the liberated p-nitroanilide at 405 nm and estimated from the decrease in the rate of digestion caused by 10 μM thiorphan, a specific inhibitor of neprilysin. Protein concentrations were determined using a BCA protein assay kit (Pierce).

### Western blot

For PGRN protein analysis, supernatants were mixed with equal amount of in 2× sample buffer boiled for 5 min, and resolved on a 10% SDS-PAGE gel. Proteins were transferred onto a nitrocellulose membrane and blocked over-night with membrane blocking agent (GE Healthcare) at 4°C. The blots were incubated in PBST with 1:250 anti-mouse PGRN polyclonal antibody (R&D Systems AF2557) for 1hour followed by extensive washing. After incubating with horseradish peroxidase-conjugated anti-sheep IgG secondary antibody (R&D Systems HAF016; 1:4,000) at room temperature for 1 hour, blots were visualized using enhanced chemiluminescence (GE Healthcare) and a Bio-Rad ChemiDoc MP Image System (Bio-Rad Laboratories, ON Canada). The same blot was stained with mouse monoclonal β-actin antibody (Sigma AC-40; 1:1000), as a control for total protein loading.

### Quantitative analysis

Surveys of Aβ deposition were performed in a 100X field in six 30μm serial anterior-posterior coronal sections, through each of three key brain regions, the frontal cortex, hippocampus, and entorhinal cortex. For quantitative assessment, the total area occupied by anti-Aβ immunoreactive deposits was measured and amyloid burden calculated as the percent of area in the measurement field occupied by reaction product. For denstiometric analyses of progranulin and synaptophysin immunolabeling in the hippocampus and cortex, a sampling frame of x = 50 μm, y = 50 μm, and z = 20 μm was used to sample these regions in each of 3 coronal sections. Optical densitometry was performed using Axiovision and Zen Pro software (Carl Zeiss). Digital images were captured on a Zeiss AxioObserver.Z1 microscope at a 20x magnification using a Zeiss Axiocam 506 monochrome camera. For comparisons of staining intensity, all images were collected using identical exposure settings using the same illumination intensity and filters. Measured values were corrected for non-specific background staining by subtracting values obtained from negative controls. Unbiased stereological measurements were obtained using a computer-assisted image analysis system and Zeiss Axiovision 4.3 image analysis software. The investigator was blinded to treatment condition.

### Statistical analysis

Data were analyzed using an analysis of variance. Where significant F-values were obtained, planned pair-wise comparisons were made using Newman-Keuls. Differences were considered statistically significant when *p* < 0.05. For ELISAs, data were analyzed using Mann-Whitney non-parametic statistics.

## Results

### Intrahippocampal infusion of ND-602 increases PGRN expression in the hippocampus

**Fig 2:**
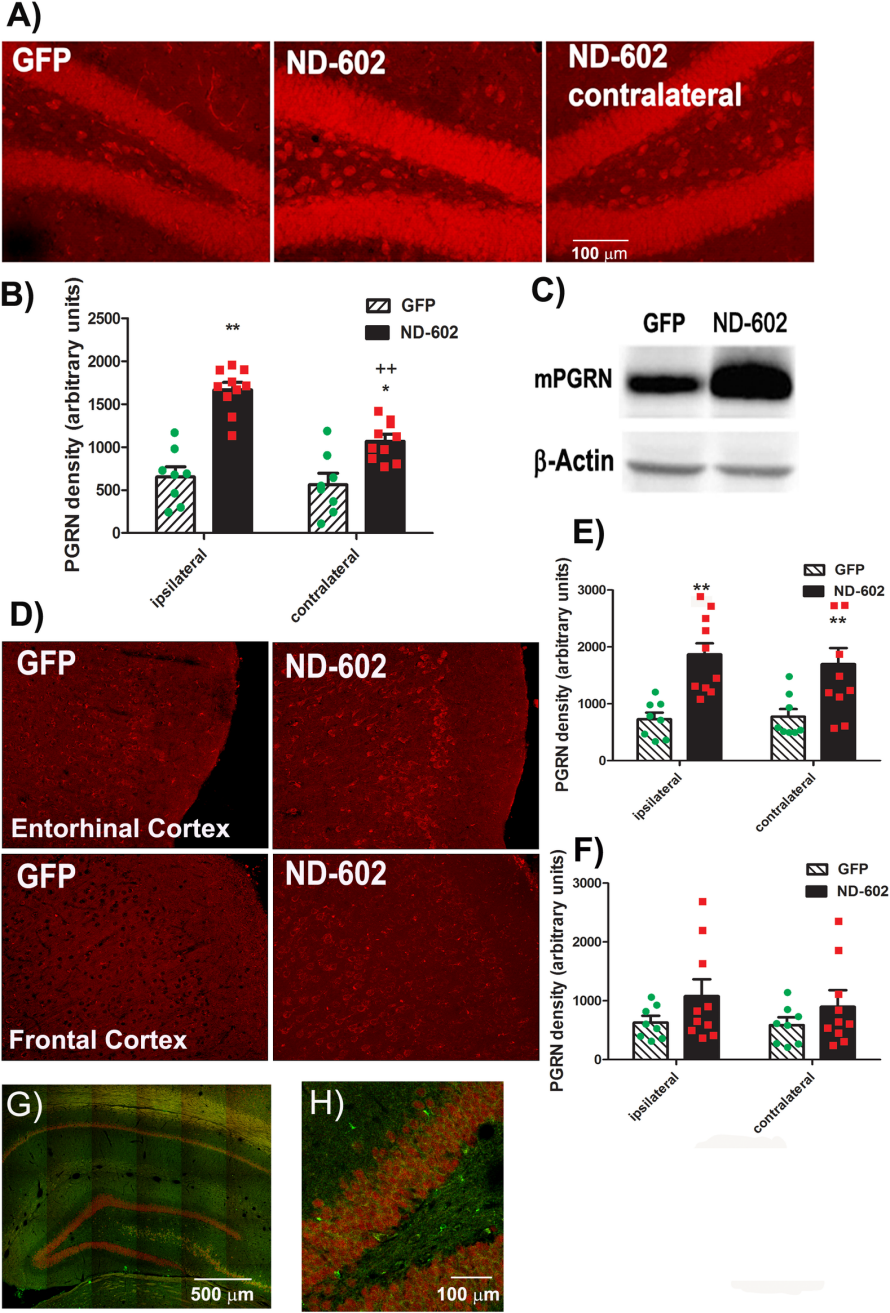
PGRN immunolabeling following ND-602. **(A)** Representative photomicrographs depicting PGRN immunolabeling in the dentate gyrus of the Tg2576 mouse brain following unilateral intracerebral administration of either LV-GFP, or LV-PGRN (ND-602). **(B)** The hippocampal density of PGRN immunolabeling was significantly elevated following ND-602 administration in both the ipsilateral and contralateral hemispheres. **(C)** PGRN protein expression (progranulin/β-actin gray values) in the hippocampus was detected by western blot assay. **(D)** Representative fluorescent photomicrographs depicting PGRN immunolabeling in the entorhinal cortex and frontal cortex following unilateral intracerebral administration of either LV-GFP, or LV-PGRN (ND-602). (E) The density of PGRN immunolabeling was significantly elevated following ND-602 administration in both hemispheres. By contrast, elevations in the **(F)** frontal cortex did not reach statistical significance. (G) Representative fluorescent photomicrograph depicting GFP (green) and NeuN (red) immunolabeling throughout the ipsilateral hippocampus following lentiviral delivery. The image is a tiled composite of multiple images obtained at 20X magnification. A closer view, (H) shows viral transduction of both NeuN-positive and–negative cells. Each bar represents the mean (± S.E.M.) (*n* = 8–10) optical density measured across 4 coronal sections. ** sig. diff. from GFP-treated controls, *p* < 0.001; * *p* < 0.05. ++ sig. diff. from contralateral hemisphere, *p* < 0.001.

The use of ND-602 has been used previously to enhance PGRN expression in nigrostriatal neurons in a mouse model of Parkinson’s disease [23]. In order to assess the efficacy of this gene delivery approach for hippocampal transduction, we examined immunolabeling for the target protein, PGRN, in the hippocampus approximately 4 months following unilateral intrahippocampal infusion of the lentiviral construct, ND-602. We found that *in vivo* gene transfer by ND-602 resulted in detectable elevations in PGRN immunolabeling ipsilateral to the site of ND-602 infusion, as compared to that seen following lentiviral delivery of GFP alone (Fig 2). PGRN immunolabeling in the contralateral hippocampus was also significantly elevated, though immunolabeling density was significantly lower than in the ipsilateral hemisphere. Elevations in PGRN immunlabeling density were also detected in the more distal region, the entorhinal cortex. Although PGRN levels also appeared to be elevated in the frontal cortex, variability was high, preventing the data from achieving statistical significance. These results indicate that ND-602 effectively transfects hippocampal cells to induce the expression of PGRN within cells of the hippocampus. This is consistent with previous reports of the same viral vector inducing PGRN expression within cells of the substantia nigra [23]. Double-labeling with the neuronal marker, NeuN, indicated effective transduction of both neuronal and non-neuronal cells.

### Intrahippocampal infusion of ND-602 reduces the appearance of amyloid plaques in the hippocampus

**Fig 3:**
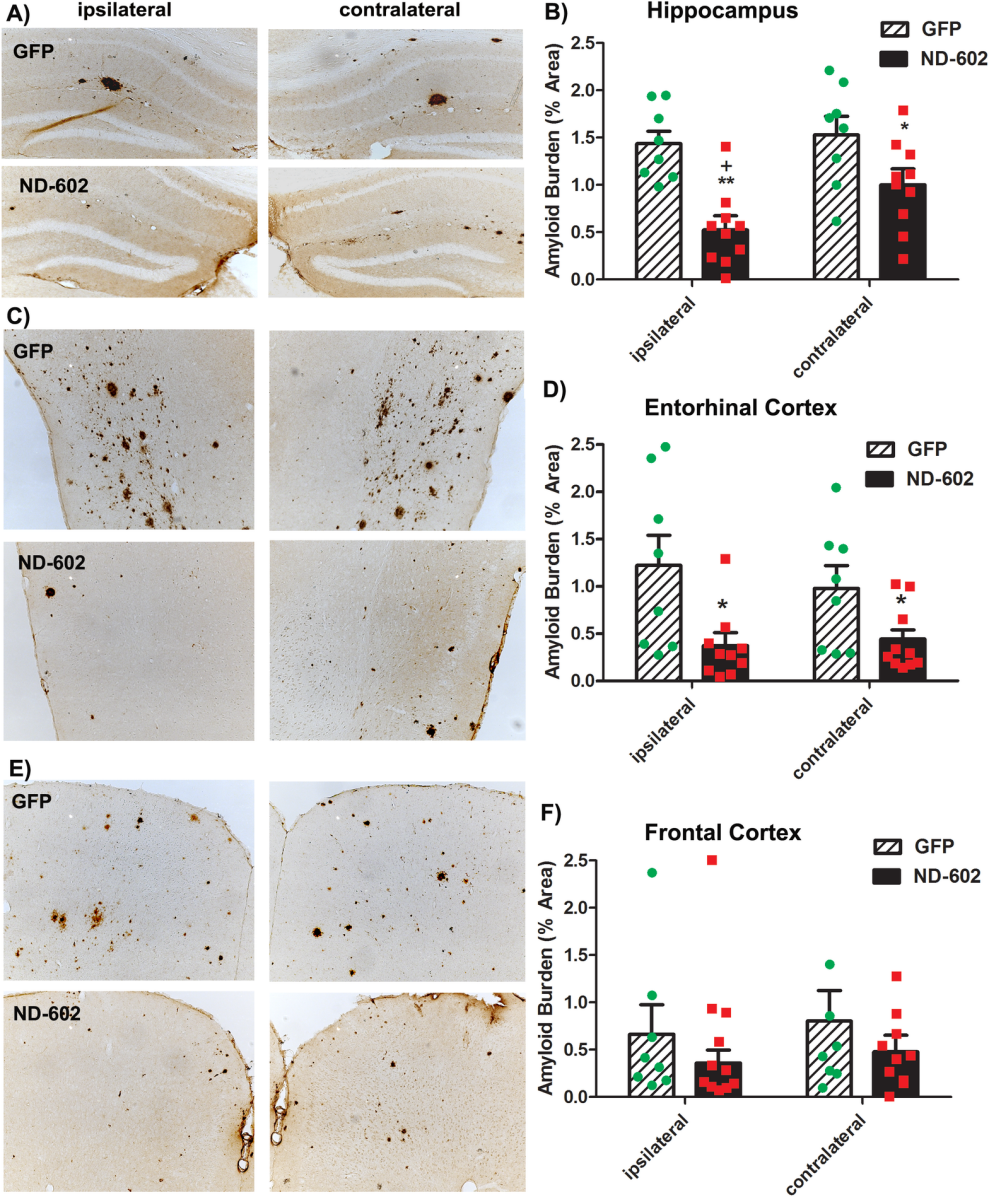
Beta-amyloid burden following ND-602. **(A,C,E)** Representative photomicrographs depicting β-amyloid immunolabeling in the **(A)** hippocampus, **(C)** entorhinal cortex, and **(E)** frontal cortex of a Tg2576 mouse brain following unilateral intracerebral administration of either LV-GFP, or LV-PGRN (ND-602). Amyloid burden was significantly reduced in the **(B)** dentate gyrus and **(D)** entorhinal cortex of those animals receiving ND-602 administration. **(F)** Apparent reductions in amyloid burden observed in the frontal cortex failed to reach statistical significance due to a high degree of variability in deposition in this region, at this time point. Each bar represents the mean (± S.E.M.) (*n* = 8–10) amyloid burden (% area) measured across 4 coronal sections. ** sig. diff. from GFP-treated controls, *p* < 0.001; * *p* < 0.05 + sig. diff. from contralateral hemisphere, *p* < 0.05.

By 12 months of age, substantial amyloid pathology typically develops in these mice [20]. To assess the influence of ND-602 on the development of these plaques, immunocytochemical analysis of burden was performed using a pan-Aβ antibody. Quantitative analysis of multiple immunostained sections revealed the appearance of Aβ immunoreactive deposits in both the hippocampus and frontal cortex and entorhinal cortex of these transgenic mice. In those animals having received intrahippocampal infusion of ND-602, a significant decline in amyloid burden was evident in the ipsilateral hippocampus (Fig 3). Consistent with the induction of PGRN expression in both hemispheres, amyloid burden was also significantly reduced in the contralateral hemisphere. In the entorhinal cortex, another region typically characterized by amyloid pathology in both human AD and animal models, ND-602 significantly reduced amyloid burden in both hemispheres. In the frontal cortex, however, the appearance of amyloid deposition was more variable. Although ND-602 did tend to reduce the appearance of amyloid in this region, the high degree of variability limited statistical power and significance was not achieved.

**Fig 4:**
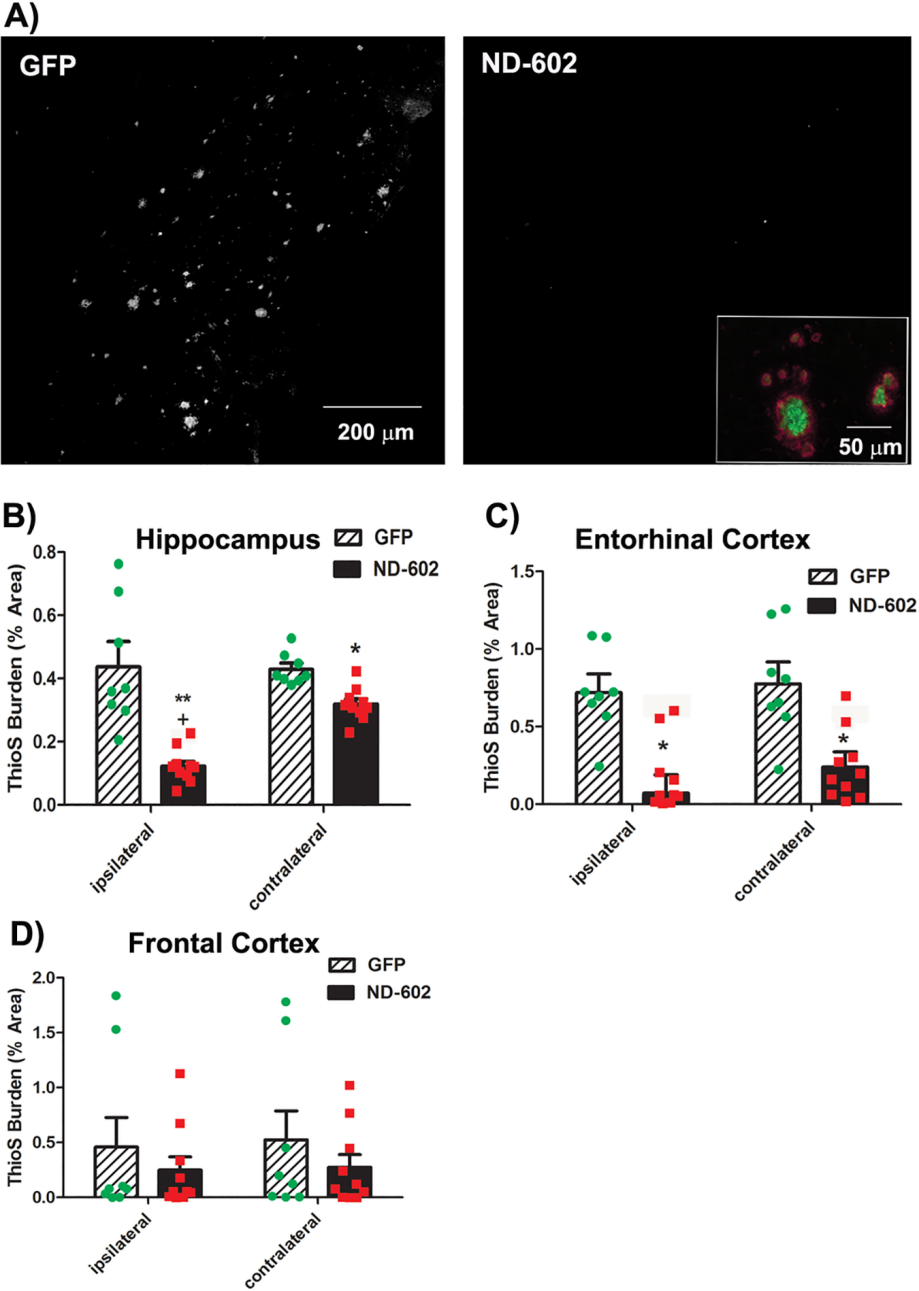
Plaque burden following ND-602. **(A)** Representative photomicrographs depicting ThioS staining in the entorhinal cortex of a Tg2576 mouse brain following unilateral intracerebral administration of either LV-GFP or LV-PGRN (ND-602). Inset depicts β-amyloid immunolabeling (red) and ThioS staining (green) of a representative plaque in the hippocampal dentate gyrus of a Tg2576 mouse brain at 12 months of age. ThioS (plaque) burden was significantly reduced in both the ipsilateral and contralateral **(B)** dentate gyrus and **(C)** entorhinal cortex of those animals receiving ND-602 administration. Reductions in the appearance of plaques in the **(D)** frontal cortex did not reach statistical significance. Each bar represents the mean (± S.E.M.) (*n* = 8–10) amyloid burden (% area) measured across 4 coronal sections. ** sig. diff. from GFP-treated controls, *p* < 0.001; * *p* < 0.05 + sig. diff. from contralateral hemisphere, *p* < 0.05.

AD neuropathology has been associated primarily with plaques containing Aβ peptide that has assembled into crossed β-fibrils [24]. In order to identify changes in this type of Aβ deposit, we performed fluorescent double-label immunohistochemistry using an anti-Aβ antibody and Thioflavine-S (ThioS), known to bind fibrillar Aβ. Using this approach, ThioS staining was found throughout the hippocampus, frontal cortex and entorhinal cortex of these Tg2576 mice. In those animals having received ND-602, a significant decline in ThioS staining was evident in the both the ipsilateral and contralateral hippocampus (Fig 4). Significant reductions in plaque burden were also evident in the entorhinal cortex, while ThioS staining in the frontal cortex was highly variable in these mice and reductions failed to reach statistical significance. Having both markers in the same tissue permitted direct comparisons of diffuse plaques (Aβ-positive, ThioS-negative) and those containing fibrillar Aβ (Aβ-positive, ThioS-positive). Immunostaining for Aβ revealed more deposits than ThioS staining and all ThioS-positive deposits were also Aβ-positive. We found that approximately 68, 71, and 81% of Aβ-positive deposits were also stained with ThioS in the frontal cortex, entorhinal cortex, and hippocampus, respectively. The proportion of Aβ-immunostaining occupied by ThioS staining was not altered by ND-602 treatment, suggesting plaque subtypes were not differentially regulated.

### ND-602 increases neprilysin immunoreactivity and activity

**Fig 5:**
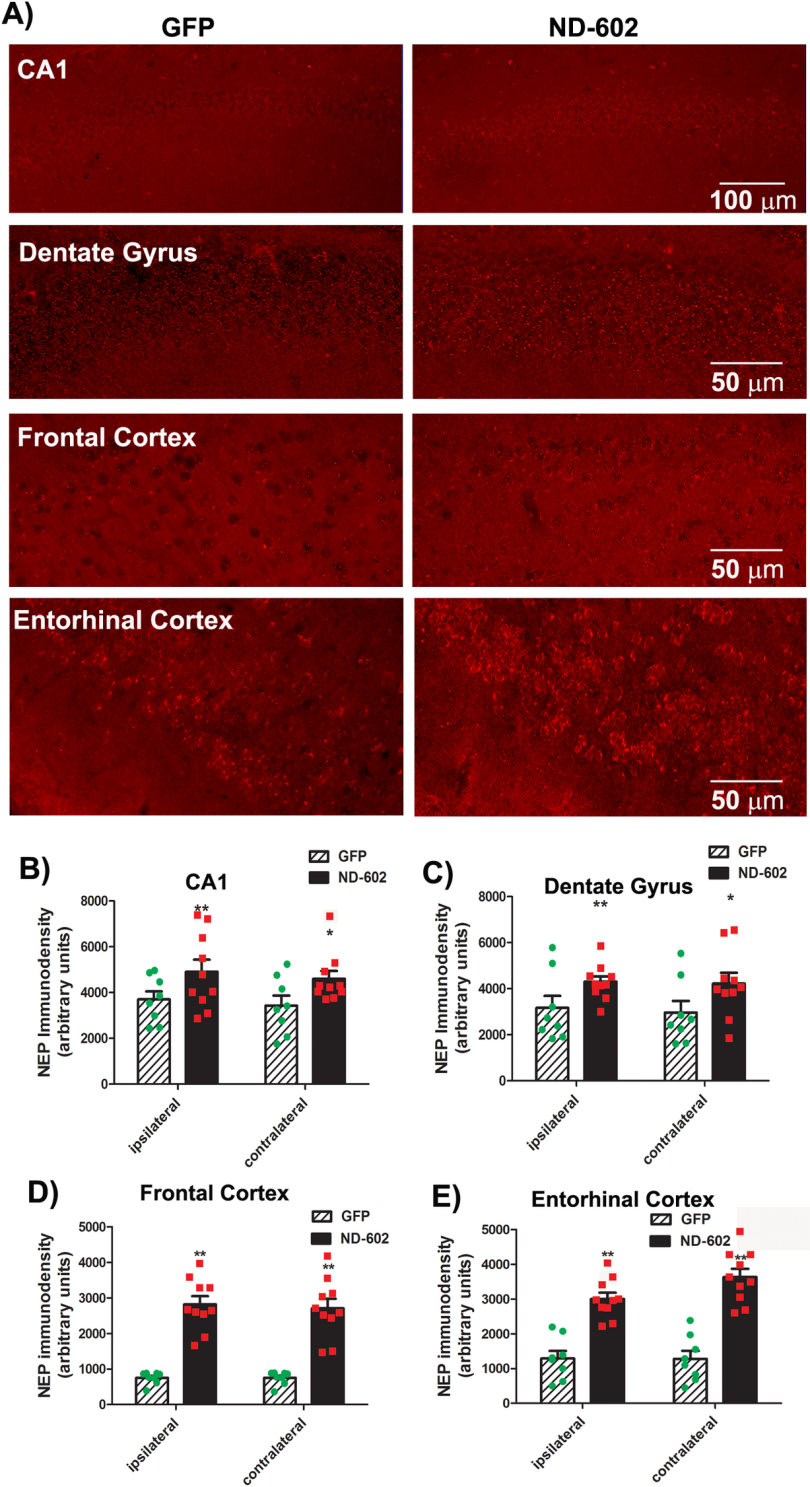
Neprilysin immunolabeling following ND-602. **(A)** Representative fluorescent photomicrographs depicting neprilysin (NEP) immunolabeling in the ipsilateral CA1, dentate gyrus, frontal cortex, and entorhinal cortex of a Tg2576 mouse brain following unilateral intracerebral administration of LV-GFP, or LV-PGRN (ND-602). The density of NEP immunolabeling was significantly elevated following ND-602 administration in the **(B)** CA1, **(C)** dentate gyrus, **(D)** frontal cortex, and **(E)** entorhinal cortex, both the ipsilateral and contralateral hemispheres. Each bar represents the mean (± S.E.M.) (*n* = 8–10) optical density measured across 4 coronal sections. ** sig. diff. from GFP-treated controls, *p* < 0.001; * *p* < 0.05.

**Fig 6:**
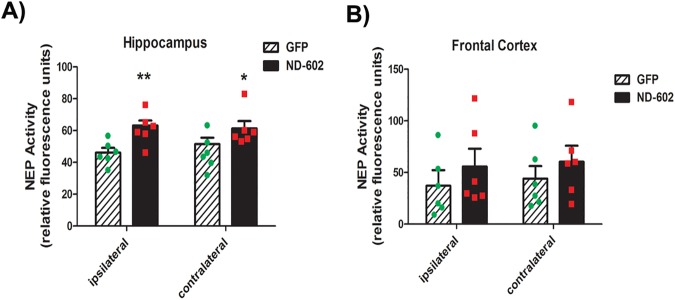
Neprilysin activity following ND-602. The neprilysin-dependent neutral endopeptidase activity was elevated in the **(A)** hippocampus and **(B)** frontal cortex following LV-PGRN (ND-602) administration. Each bar represents the mean (± S.E.M.) (*n* = 6) specific neprilysin activity, expressed as nmols/min/mg protein. ** sig. diff. from GFP-treated controls, *p* < 0.001

Neprilysin (NEP) is the primary Aβ-degrading enzyme in the brain[25] and strategies aimed at enhancing NEP activity may be of therapeutic advantage for AD[26, 27]. Here, we observed changes in NEP following ND-602. The immunodensity of NEP was significantly elevated in both hemispheres of the DG and CA1 region of the hippocampus of those animals treated with ND-602 (Fig 5). Elevations in NEP immunolabeling were also detected in the entorhinal cortex and frontal cortex. A NEP activity assay revealed that intrahippocampal infusion of ND-602 triggers significant alterations in NEP specific activity in both the hippocampus and frontal cortex (Fig 6).

### ND-602 reduces inflammation

**Fig 7:**
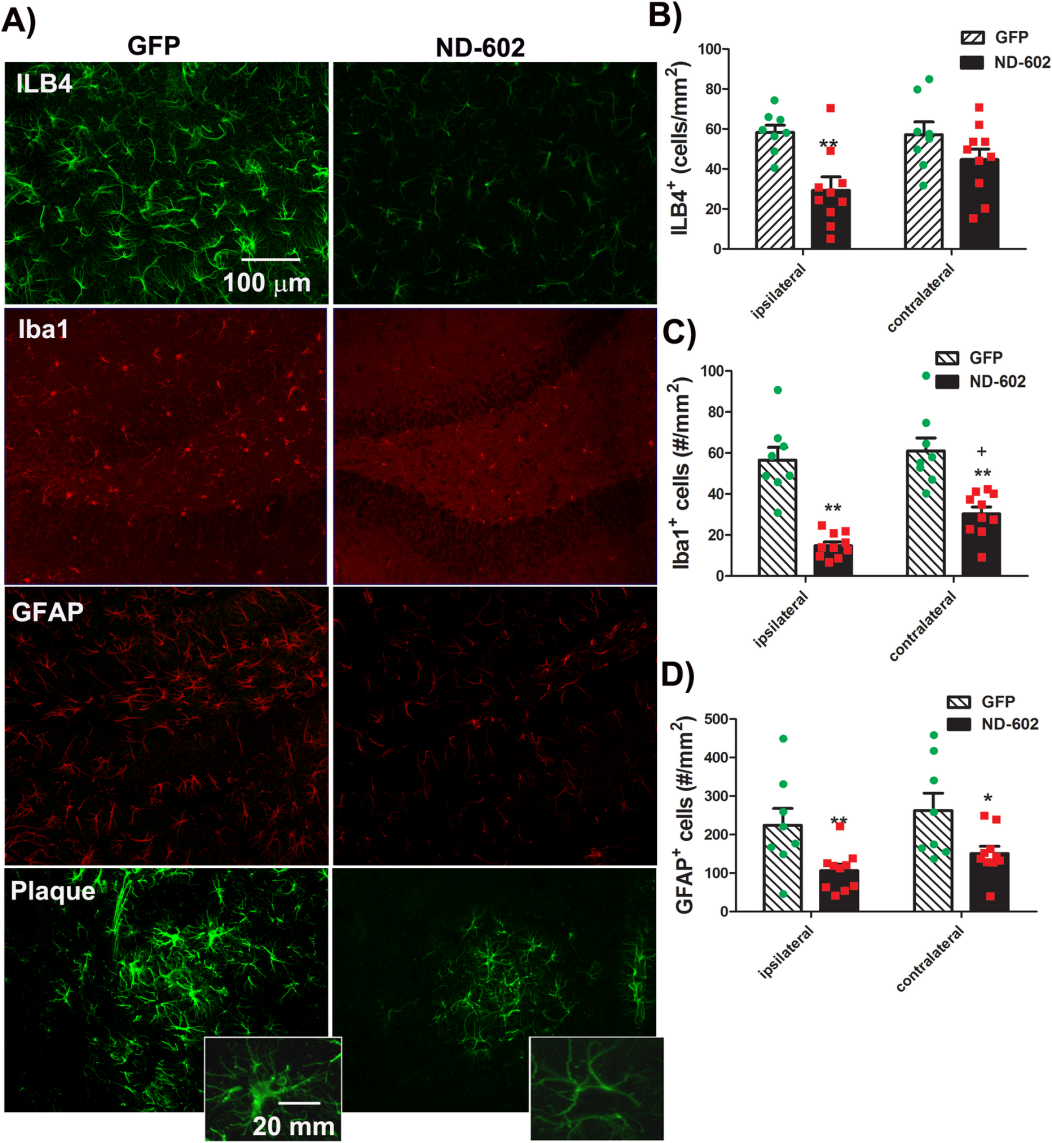
Microglial cell counts in the hippocampus following ND-602. **(A)** Representative fluorescent photomicrographs depicting ILB4, Iba1, and GFAP staining in the hippocampusfollowing lentiviral delivery of LV-GFP or LV-PGRN (ND-602). ILB4 staining surrounding a hippocampal plaque is also depicted in the lower panels. Insets depict magnified view of an individual microglial cell. Neuroinflammation, as evidenced by **(B)** ILB4 staining and **(C)** Iba1 immunolabeling of microglial cells, was significantly reduced in those animals who received unilateral intracerebral administration of ND-602. **(D)** Similar reductions in the astrocytic marker, GFAP, were also observed following treatment with ND-602. Each bar represents the mean (± S.E.M.) (*n* = 8–10) density (#/mm2) of ILB4^+^, Iba1^+^, or GFAP^+^cells counted throughout the dorsal hippocampus across 4 coronal sections. ** sig. diff. from GFP-treated controls, *p* < 0.001; * *p* < 0.05.

Enhanced astrocytosis and microgliosis are characteristic features of AD pathology and the Tg2576 phenotype. Neuroinflammatory mechanisms are believed to contribute to the cascade of events leading to neuronal degeneration in AD. This makes PGRN a particularly intriguing therapeutic target, as PGRN is a potent regulator of inflammation, both in the periphery and the CNS. We were, therefore, interested to see whether elevating PGRN levels, via ND-602 treatment, would impact the microglial activation characteristically seen in Tg2576 mice. Microglial activation was assessed by two microglial markers, fluorescein-conjugated isolectin B_4_ (ILB4), and ionized calcium binding adaptor molecule 1 (Iba1). There was a significant reduction in the number of cells labeling for both ILB4 and Iba1 in the hippocampus (Fig 7). There was, similarly, a significant reduction in the number of cells immunolabeled for the astrocytic marker, glial fibrillary acidic protein (GFAP) in the hippocampus. Thus, ND-602 appears to reduce the elevation in microglial expression characteristic of AD pathology.

### ND-602 increases synaptic density in the hippocampus

**Fig 8:**
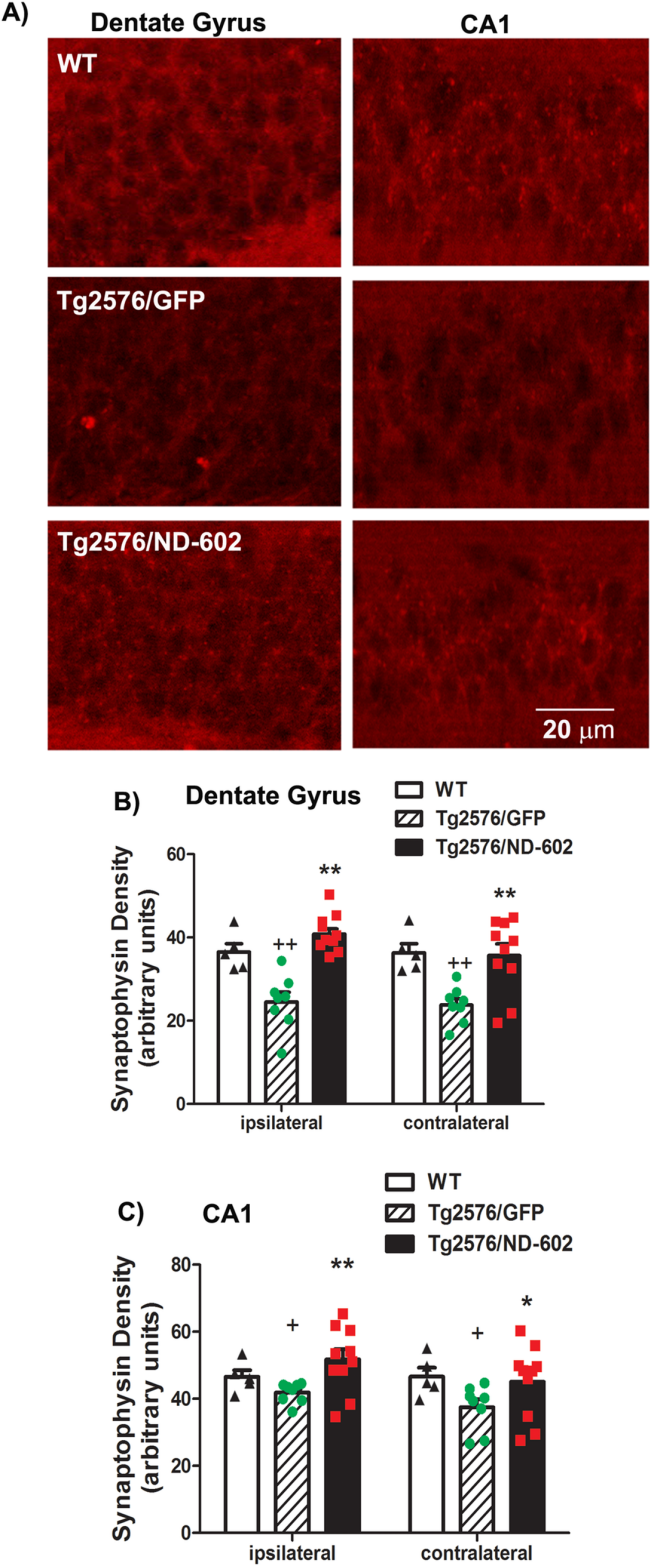
Synaptic density following ND-602. **(A)** Representative fluorescent photomicrographs depicting immunolabeling for the synaptic protein, synaptophysin, in the dentate gyrus and CA1 region of the hippocampus, following lentiviral delivery of LV-GFP or LV-PGRN (ND-602). Immunolabeling of synaptophysin was significantly reduced in the **(B)** dentate gyrus and **(C)** CA1 region of the hippocampus of Tg2576 mice, as compared to wild-type controls. Density of synaptophysin labeling was significantly increased in both regions following administration of ND-602. Each bar represents the mean (± S.E.M., *n* = 5–10) optical density measured. ** sig. diff. from GFP-treated controls, *p* < 0.001; * *p* < 0.05. + sig. diff. from wild-type controls, *p* < 0.05.

Synaptophysin is a synaptic molecule present in presynaptic terminals and a robust marker for functional neurons. It has previously been found that synapse density is decreased in the molecular layer of the dentate gyrus in Tg2576 mice at 6–9 months of age [28]. In order to assess the effect of ND-602 on the degenerative synaptic pathology characteristic of these mice, we examined immunolabeling of the synaptic protein, synaptophysin. In dentate gyrus and CA1 region of the hippocampus, transgenic animals were found to have a significant reduction in synaptophysin immunolabeling, as compared to age-matched C57BL6 wild-type controls (Fig 8). This synaptic atrophy was most apparent in the molecular layer of the dentate gyrus. However, treatment with ND-602 significantly elevated synaptophysin density in both the dentate gyrus and CA1 region. Thus, ND-602 appears to prevent the loss of synaptic density that typically occurs in Tg2576 mice.

## Discussion

Progranulin is a secreted cysteine-rich protein with a molecular weight of 90 KDa in its glycosylated form. Biological activity has been ascribed to both the intact progranulin protein and the 6-KDa peptides (granulin domains) that result from post translational processing of the intact protein. Progranulin is expressed by several cell types and it has modulatory roles in normal and pathophysiological processes such as blastocyst formation, wound healing, inflammation and tumorigenesis[29]. PGRN is widely distributed throughout the CNS where it is found primarily in neurons and microglia but has also been detected, at much lower levels, in astrocytes and oligodendrocytes [10, 30]. Its functions in the adult CNS have only recently been investigated and a receptor has not yet been described. However, progranulin is known to stimulate survival signaling pathways, including both the mitogen activated protein kinase (MAPK) pathway and the phosphatidyl inositol-3 kinase kinase (PI-3K) pathway in cell lines of extraneural origin[31, 32]. These effects are independent of the presence of the insulin-like growth factor I receptor, which differentiates progranulin from conventional growth factors[31]. Recent work has demonstrated an endocytic pathway targeting extracellular progranulin to lysosomal localization mediated through the single-pass transmembrane protein, sortilin[33]. Other progranulin-triggered events, such as neuronal outgrowth, appear to be independent of sortilin[34]

Progranulin truly is a multi-function protein, being implicated in embryogenesis, wound repair, inflammation, and cell growth and survival. In mice, loss of PGRN exaggerates indices of an ageing brain, dysregulates inflammatory responses, increases susceptibility to cytotoxic stresses, reduces synaptic connectivity and impairs plasticity [6, 35, 36]. Consistent with its strong immunoreactivity in activated microglia[1], PGRN is known to be a critical regulator of CNS inflammation[2, 6, 17]. This may explain reports of upregulated expression in numerous disease states involving microglial activation, including motor neuron disease, lysosomal storage disease, and Alzheimer’s disease [1, 3, 4, 37]. It is now understood that PGRN functions as an autocrine neuronal growth factor, important for long-term neuronal survival [2, 38], which suggests that PGRN has the potential to influence susceptibility to a wide range of neurodegenerative diseases, including AD.

Here, we have demonstrated that injection of lentiviral vectors expressing mouse PGRN effectively elevates PGRN expression in the hippocampus, resulting in a significant reduction in amyloid plaque burden in the Tg2576 mouse model of AD. This is consistent with previously reported findings of a negative correlation between hippocampal PGRN levels and plaque load in 5xFAD mice following lentiviral PGRN overexpression, along with reduced hippocampal neuron loss [16]. Although Tg2576 mice do not display hippocampal neuron loss, they do exhibit synaptic atrophy, which we found to be significantly attenuated following PGRN overexpression. Unique to this study, is the discovery of elevated neprilysin activity following lentiviral PGRN delivery, a possible mechanism of action for the observed reductions in plaque burden.

Impaired clearance of Aβ contributes significantly to the abnormal accumulation and aggregation of Aβ that characterizes AD pathology[39]. Neprilysin, is an Aβ-degrading metalloendopeptidase, shown to efficiently degrade Aβ[40], both monomeric and oligomeric forms, and has been identified as a potential therapeutic target for the treatment of AD. Indeed, NEP expression has been inversely associated with vulnerability to amyloid deposition in AD patients[41], with regions of lower expression displaying greater levels of amyloid pathology. Expression levels of NEP has been shown to decline with age, in association with a decline in Aβ clearance[42] In mice, NEP deficiency results in a gene dose-dependent increase in levels of Aβ40 and Aβ42 in the brain[42] while the induction of NEP expression by viral vector transfer in APP transgenic mice [43], or convection enhanced delivery in aged rats [44] has been shown to result in a reduction of Aβ accumulation and behavioral deficits. The level of NEP is reduced in AD patients[45] and aging transgenic AD mice[46]. Elevating NEP expression reduces neurodegenerative pathology and improves cognitive performance in AD mice.[43] Here, we have demonstrated that enhancing PGRN expression, through viral vector delivery, results in an increase in NEP expression and activity. This change in NEP may be a key factor in the reduction in amyloid plaque burden observed in these animals following ND-602.

Neuroinflammation is another key feature of AD pathology[47, 48], one that is replicated in transgenic mouse models, including Tg2576 mice[49–51]. Consistent with its robust expression in microglia, PGRN serves as a regulator of neuroinflammation [30, 36] through both anti-inflammatory and pro-inflammatory processes. Macrophages deficient in PGRN show decreased secretion of the anti-inflammatory cytokine interleukin-10, with a concomitant increase in the secretion of inflammatory cytokines such as interleukin-6 and TNF-α [6]. PGRN directly binds to TNFR and blocks the binding of TNF-α to its receptors, providing one potential molecular mechanism underlying PGRN-mediated anti-inflammation[52]. PGRN deficient mice display a dysregulated immune response in the brain, with a more pronounced age-dependent increase in glial activation [5, 6] and highly exaggerated inflammatory responses to various triggers, including LPS and bacterial infection [6]. Furthermore, microglia cultured from these mice were found to have toxic effects on co-cultured neurons [6]. Conversely, mice overexpressing PGRN display reduced pro-inflammatory cytokine release and elevated anti-inflammatory cytokine release in response to LPS [53]. Here, we describe hippocampal microgliosis observed in Tg2576 mice, which was significantly blunted by ND-602-induced elevations in PGRN. With inflammation playing such a significant role in the development of Aβ pathology and subsequent toxicity[54, 55], the neuroprotective actions of ND-602, reported here, could result, at least in part, from regulation of inflammatory processes by PGRN.

While the deposition of amyloid is a key feature of AD pathology, it is synaptic atrophy that is most likely responsible for the ensuing cognitive deficits. Indeed, cognitive dysfunction develops prior to the appearance of plaques, with a rather poor correlation between amyloid burden and cognitive function. Synapse loss is an early event in the disease process and serves as a structural correlate involved in cognitive decline [56, 57]. While Tg2576 mice do not exhibit global neuronal cell loss [28, 58], a decline in synapse density is a characteristic feature of this model [28, 59]. PGRN is known to function as an autocrine neurotrophic factor, secreted from neurons in an activity-dependent manner, to promote neuronal survival and synapse formation[7, 60, 61]. While PGRN deficiency leads to a reduction in synaptic density and function [60, 61], the enhancement of PGRN expression, as described here, results in an increase in hippocampal synaptic density.

The multi-functional nature of PGRN makes it difficult to zero in on the precise mechanism of action at play here. Certainly, a reduction in amyloid burden by PGRN could explain the preservation of hippocampal synaptic density through a reduction in synaptoxicity caused by Aβ. However, PGRN could also play a more direct role as a neurotrophic factor, promoting neuroplasticity and neuroprotection. PGRN has been shown to regulate neuronal outgrowth and branching [34, 62], through phosphorylation of glycogen synthase kinase 3β (GSK-3β), a well-known substrate of the serine/ threonine kinase AKT1/PKBa. Phosphorylation of GSK3β at the S9 residue is critical for its inactivation[63] and such regulation of the Akt/GSK-3β signaling pathway promotes synaptogenesis and axon growth [64–66]. PGRN may also be actively protecting against synaptic atrophy through prevention of excitotoxicity, a key event in AD pathogenesis [67]. In cultures of cortical neurons, PGRN has been shown to protect against glutamate toxicity [53, 68] and mice overexpressing PGRN show reduced infarct size and functional deficits in an ischemic model [53]. In light of its role in AD pathogenesis, modulation of excitotoxicity may be one means by which ND-602 protects against neuronal atrophy. Another hallmark feature of AD pathogenesis is oxidative stress [69]. Activation of the PI_3_K/AKT signaling pathway is also involved in the regulation of cellular apoptosis under oxidative stress [70–73], making this pathway a good therapeutic target for oxidative stress-related neurodegenerative disease, such as AD. PGRN has been demonstrated to activate the PI_3_K/AKT pathway in primary neuronal cultures, resulting in protection against oxidative stresses triggered by H_2_O_2_ [68]. Conversely, depletion of PGRN renders primary neurons more vulnerable to both oxidative stress and excitotoxicity [74].

Synaptic density can also be influenced by Wnt signaling. Wnt proteins are evolutionarily conserved secreted glycolipoproteins that play an important role in mediating cell proliferation, differentiation, cell fate determination during embryonic development and tissue homeostasis in the central nervous system in adult [75–78]. Wnt signaling pathways may play a critical role in determining the balance between neuronal survival and death in degenerative disease [38, 79]. Wnt signaling also regulates the expression of other growth factors, such as BDNF[80], whose expression is reduced by Aβ [81, 82]. BDNF can increase synaptic plasticity and neurogenesis and promotes neural functional recovery and synaptophysin expression [83]. Blocking Wnt signaling triggers synaptic degeneration, while activation of this pathway restores functional circuits in the adult hippocampus[84]. Mice deficient in PGRN show altered Wnt signaling, suggesting a role for PGRN in regulating this signaling pathway [85].

As mentioned, PGRN regulates GSK3β [86, 87], which is highly expressed in the brain and has been identified as the principal kinase responsible for the hyperphosphorylation of tau in AD [41] [42] and modulates the generation of Aβ[88], playing an important role in the pathogenesis of AD. Several findings have suggested that GSK3 activity might be increased in AD[89] and inhibitors of GSK3β reduce amyloid deposition and improve cognitive function in models of AD [90–92]. The increase in PGRN expression induced by ND-602 in this study could have reduced amyloid plaque burden through phosphorylation of GSK3β, thereby reducing its activity.

In summary, ND-602, a lentiviral construct for the targeted expression of PGRN, effectively reduced amyloid plaque burden in the Tg2576 mouse model of AD. This was accompanied by a reduction in inflammation and attenuation of the deficits in synaptic density observed in this model. As a widely expressed multifunctional protein, PGRN regulates a diverse series of cellular processes involved in Alzheimer's disease (AD) pathology, making it a good therapeutic target.

## Supporting information

S1 Appendix Full pLenti6/V 5-mGranulin vector sequence. (PDF)
