# Supplementary material for: Progranulin gene delivery reduces plaque burden and synaptic atrophy in a mouse model of Alzheimer's disease
Source: PLoS One. 2017 Aug 24;12(8):e0182896. doi: 10.1371/journal.pone.0182896 (PMC5570501; doi:10.1371/journal.pone.0182896)
Supplement: S1 Appendix — (PDF) [file pone.0182896.s001.pdf]

pLenti6/V 5-mGranulin vector Sequence:

1 aagggctcga gtctagaggg cccgcggttc gaaggtaagc ctatccctaa

51 ccctctcctc ggtctcgatt ctacgcgtac cggttagtaa tgagtttgga

101 attaattctg tggaatgtgt gtcagttagg gtgtggaaag tccccaggct

151 ccccaggcag gcagaagtat gcaaagcatg catctcaatt agtcagcaac

201 caggtgtgga aagtccccag gctccccagc aggcagaagt atgcaaagca

251 tgcatctcaa ttagtcagca accatagtcc cgcccctaac tccgcccatc

301 ccgcccctaa ctccgcccag ttccgcccat tctccgcccc atggctgact

351 aattttttt atttatgcag aggccgaggc cgcctctgcc tctgagctat

401 tccagaagta gtgaggaggc tttttggag gcctaggctt ttgcaaaaag

451 ctcccgggag cttgtatatc cattttcgga tctgatcagc acgtgttgac

501 aattaatcat cggcatagta tatcggcata gtataatacg acaaggtgag

551 gaactaaacc atggccaagc ctttgtctca agaagaatcc accctcattg

601 aaagagcaac ggctacaatc aacagcatcc ccatctctga agactacagc

651 gtcgccagcg cagctctctc tagcgacggc cgcatcttca ctggtgtcaa

701 tgtatatcat tttactgggg gaccttgtgc agaactcgtg gtgctgggca

751 ctgctgctgc tgcggcagct ggcaacctga cttgtatcgt cgcgatcgga

801 aatgagaaca ggggcatctt gagcccctgc ggacggtgcc gacaggtgct

851 tctcgatctg catcctggga tcaaagccat agtgaaggac agtgatggac

901 agccgacggc agttgggatt cgtgaattgc tgccctctgg ttatgtgtgg

951 gagggctaag cacaattcga gctcggtacc tttaagacca atgacttaca

1001 aggcagctgt agatcttagc cactttttaa aagaaaaggg gggactggaa

1051 gggctaattc actcccaacg aagacaagat ctgctttttg cttgtactgg

1101 gtctctctgg ttagaccaga tctgagcctg ggagctctct ggctaactag

1151 ggaacccact gcttaagcct caataaagct tgccttgagt gcttcaagta

1201 gtgtgtgccc gtctgttgtg tgactctggt aactagagat ccctcagacc

1251 cttttagtca gtgtggaaaa tctctagcag tagtagttca tgtcatctta

1301 ttattcagta tttataactt gcaaagaaat gaatatcaga gagtgagagg

1351 aacttgttta ttgcagctta taatggttac aaataaagca atagcatcac

1401 aaatttcaca aataaagcat ttttttcact gcattctagt tgtggtttgt

1451 ccaaactcat caatgtatct tatcatgtct ggctctagct atcccgcccc

1501 taactccgcc catcccgccc ctaactccgc ccagttccgc ccattctccg

1551 ccccatggct gactaatttt ttttatttat gcagaggccg aggccgcctc

1601 ggcctctgag ctattccaga agtagtgagg aggctttttt ggaggcctag

1651 ggacgtaccc aattcgccct atagtgagtc gtattacgcg cgctcactgg

1701 ccgtcgtttt acaacgtcgt gactgggaaa accctggcgt tacccaactt

1751 aatcgccttg cagcacatcc ccctttcgcc agctggcgta atagcgaaga

1801 ggcccgcacc gatcgccctt cccaacagtt gcgcagcctg aatggcgaat

1851 gggacgcgcc ctgtagcggc gcattaagcg cggcgggtgt ggtggttacg

1901 cgcagcgtga ccgctacact tgccagcgcc ctagcgcccg ctcctttcgc

1951 tttcttccct tcctttctcg ccacgttcgc cggctttccc cgtcaagctc

2001 taaatcgggg gctcccttta gggttccgat ttagtgcttt acggcacctc

2051 gaccccaaaa aacttgatta gggtgatggt tcacgtagtg ggccatcgcc

2101 ctgatagacg gtttttcgcc ctttgacgtt ggagtccacg ttctttaata

2151 gtggactctt gttccaaact ggaacaacac tcaaccctat ctcggtctat

2201 tcttttgatt tataagggat tttgccgatt tcggcctatt ggttaaaaaa

2251 tgagctgatt taacaaaaat ttaacgcgaa ttttaacaaa atattaacgc

2301 ttacaattta ggtggcactt ttcggggaaa tgtgcgcgga Acccctattt

2351 gtttattttt ctaaatacat tcaaatatgt atccgctcat gagacaataa

2401 ccctgataaa tgcttcaata

atattgaaaa aggaagagta tgagtattca

2451 acatttccgt gtcgccctta ttcccttttt tgcggcattt tgccttcctg

2501 tttttgctca cccagaaacg ctggtgaaag taaaagatgc tgaagatcag

2551 ttgggtgcac gagtgggtta catcgaactg gatctcaaca gcggtaagat

2601 ccttgagagt tttcgccccg aagaacgttt tccaatgatg agcactttta

2651 aagttctgct atgtggcgcg gtattatccc gtattgacgc cgggcaagag

2701 caactcggtc gccgcataca ctattctcag aatgacttgg ttgagtactc

2751 accagtcaca gaaaagcatc ttacggatgg catgacagta agagaattat

2801 gcagtgctgc cataaccatg agtgataaca ctgcggccaa cttacttctg

2851 acaacgatcg gaggaccgaa ggagctaacc gcttttttgc acaacatggg

2901 ggatcatgta actcgccttg atcgttggga accggagctg aatgaagcca

2951 taccaaacga cgagcgtgac accacgatgc ctgtagcaat ggcaacaacg

3001

ttgcgcaaac tattaactgg cgaactactt actctagctt cccggcaaca

3051 attaatagac tggatggagg cggataaagt tgcaggacca cttctgcgct

157

3101 cggcccttcc ggctggctgg tttattgctg ataaatctgg agccggtgag

3151 cgtgggtctc gcggtatcat tgcagcactg gggccagatg gtaagccctc

3201 ccgtatcgta gttatctaca cgacggggag tcaggcaact atggatgaac

3251 gaaatagaca gatcgctgag ataggtgcct cactgattaa gcattggtaa

3301 ctgtcagacc aagtttactc atatatactt tagattgatt taaaacttca

3351 tttttaattt aaaaggatct aggtgaagat cctttttgat aatctcatga

3401 ccaaaatccc ttaacgtgag ttttcgttcc actgagcgtc agaccccgta

3451 gaaaagatca aaggatcttc ttgagatcct ttttttctgc gcgtaatctg

3501 ctgcttgcaa acaaaaaaac caccgctacc agcggtggtt tgtttgccgg

3551 atcaagagct accaactctt tttccgaagg taactggctt cagcagagcg

3601 cagataccaa atactgttct tctagtgtag ccgtagttag gccaccactt

3651 caagaactct gtagcaccgc ctacatacct cgctctgcta atcctgttac

3701 cagtggctgc tgccagtggc gataagtcgt gtcttaccgg gttggactca

3751 agacgatagt taccggataa ggcgcagcgg tcgggctgaa cggggggttc

3801 gtgcacacag cccagcttgg agcgaacgac ctacaccgaa ctgagatacc

3851 tacagcgtga gctatgagaa agcgccacgc ttcccgaagg gagaaaggcg

3901 gacaggtatc cggtaagcgg cagggtcgga acaggagagc gcacgaggga

3951 gcttccaggg ggaaacgcct ggtatcttta tagtcctgtc gggtttcgcc

4001 acctctgact tgagcgtcga tttttgtgat gctcgtcagg ggggcggagc

4051 ctatggaaaa acgccagcaa cgcggccttt ttacggttcc tggccttttg

4101 ctggcctttt gctcacatgt tctttcctgc gttatcccct gattctgtgg

4151 ataaccgtat taccgccttt gagtgagctg ataccgctcg ccgcagccga

4201 acgaccgagc gcagcgagtc agtgagcgag gaagcggaag agcgcccaat

4251 acgcaaaccg cctctccccg cgcgttggcc gattcattaa tgcagctggc

4301 acgacaggtt tcccgactgg aaagcgggca gtgagcgcaa cgcaattaat

4351 gtgagttagc tcactcatta ggcaccccag gctttacact ttatgcttcc

4401 ggctcgtatg ttgtgtggaa ttgtgagcgg ataacaattt cacacaggaa

4451 acagctatga ccatgattac gccaagcgcg caattaaccc tcactaaagg

4501 gaacaaaagc tggagctgca agcttaatgt agtcttatgc aatactcttg

4551 tagtcttgca acatggtaac gatgagttag caacatgcct tacaaggaga

4601 gaaaaagcac cgtgcatgcc gattggtgga agtaaggtgg tacgatcgtg

4651 ccttattagg aaggcaacag acgggtctga catggattgg acgaaccact

4701 gaattgccgc attgcagaga tattgtattt aagtgcctag ctcgatacat

4751 aaacgggtct ctctggttag accagatctg agcctgggag ctctctggct

4801 aactagggaa cccactgctt aagcctcaat aaagcttgcc ttgagtgctt

4851 caagtagtgt gtgcccgtct gttgtgtgac tctggtaact agagatccct

4901 cagacccttt tagtcagtgt ggaaaatctc tagcagtggc gcccgaacag

4951 ggacttgaaa gcgaaaggga qaccagagga gctctctcga cgcaggactc

5001 ggcttgctga agcgcgcacg gcaagaggcg aggggcggcg actggtgagt

5051 acgccaaaaa ttttgactatg cggaggctag aaggagagag atgggtgcga

5101 gagcgtcagt attaagcggg ggagaattag atcgcgatgg gaaaaaattc

5151 ggttaaggcc agggggaaag aaaaaatata aattaaaaca tatagtatgg

5201 gcaagcaggg agctagaacg attcgcagtt aatcctggcc tgttagaaac

5251 atcagaaggc tgtagacaaa tactgggaca gctacaacca tcccttcaga

5301 caggatcaga agaacttaga tcattatata atacagtagc aaccctctat

158

5351 tgtgtgcatc aaaggataga gataaaagac accaaggaag ctttagacaa

5401 gatagaggaa gagcaaaaca aaagtaagac caccgcacag caagcggccg

5451 ctgatcttca gacctggagg aggagatatg agggacaatt ggagaagtga

5501 attatataaa tataaagtag

taaaaattga accattagga gtagcaccca

5551 ccaaggcaaa gagaagagtg gtgcagagag aaaaaagagc agtgggaata

5601 ggagctttgt tccttgggtt cttgggagca gcaggaagca ctatgggcgc

5651 agcgtcaatg acgctgacgg tacaggccag acaattattg tctggtatag

5701 tgcagcagca gaacaatttg ctgagggcta ttgaggcgca acagcatctg

5751 ttgcaactca cagtctgggg catcaagcag ctccaggcaa gaatcctggc

5801 tgtggaaaga tacctaaagg atcaacagct cctggggatt tggggttgct

5851 ctggaaaact catttgcacc actgctgtgc cttggaatgc tagttggagt

5901 aataaatctc tggaacagat ttggaatcac acgacctgga tggagtggga

5951 cagagaaatt aacaattaca caagcttaat acactcctta attgaagaat

6001 cgcaaaacca gcaagaaaag aatgaacaag aattattgga attagataaa

6051 tgggcaagtt tgtggaattg gtttaacata acaaattggc tgtggtatat

6101 aaaattattc ataatgatag taggaggctt ggtaggttta agaatagttt

6151 ttgctgtact ttctatagtg aatagagtta ggcagggata ttcaccatta

6201 tcgtttcaga cccacctccc aaccccgagg ggacccgaca ggcccgaagg

6251 aatagaagaa gaaggtggag agagagacag agacagatcc attcgattag

6301 tgaacggatc tcgacggtat cgataagctt gggagttccg cgttacataa

6351 cttacggtaa atggcccgcc tggctgaccg cccaacgacc cccgcccatt

6401 gacgtcaata atgacgtatg ttcccatagt aacgccaata gggactttcc

6451 attgacgtca atgggtggag tatttacggt aaactgccca cttggcagta

6501 catcaagtgt atcatatgcc aagtacgccc cctattgacg tcaatgacgg

6551 taaatggccc gcctggcatt atgcccagta catgacctta tgggactttc

6601 ctacttggca gtacatctac gtattagtca tcgctattac catggtgatg

6651 cggttttggc agtacatcaa tgggcgtgga tagcggtttg actcacgggg

6701 atttccaagt ctccacccca ttgacgtcaa tgggagtttg ttttggcacc

6751 aaaatcaacg ggactttcca aaatgtcgta acaactccgc cccattgacg

6801 caaatgggcg gtaggcgtgt acggtgggag gtctatataa gcagagctcg

6851 tttagtgaac cgtcagatcg cctggagacg ccatccacgc tgttttgacc

6901 tccatagaag acaccgactc tagaggatcc actagtccag tgtggtggaa

6951 ttgatccctt caccatgtgg gtcctgatga gctggctggc cttcgcggca

7001 gggctggtag ccggaacaca gtgtccagat gggcagttct gccctgttgc

7051 ctgctgcctt gaccagggag gagccaacta cagctgctgt aaccctcttc

7101 tggacacatg gcctagaata acgagccatc atctagatgg ctcctgccag

7151 acccatggcc actgtcctgc tggctattct tgtcttctca ctgtgtctgg

7201 gacttccagc tgctgcccgt tctctaaggg tgtgtcttgt ggtgatggct

7251 accactgctg cccccagggc ttccactgta gtgcagatgg gaaatcctgc

7301 ttccagatgt cagataaccc cttgggtgct gtccagtgtc ctgggagcca

7351 gtttgaatgt cctgactctg ccacctgctg cattatggtt gatggttcgt

7401 ggggatgttg tcccatgccc caggcctctt gctgtgaaga cagagtgcat

7451 tgctgtcccc atggggcctc ctgtgacctg gttcacacac gatgcgtttc

7501 acccacgggc acccacaccc tactaaagaa gttccctgca caaaagacca

7551 acagggcagt gtctttgcct ttttctgtcg tgtgccctga tgctaagacc

159

7601 cagtgtcccg atgattctac ctgctgtgag ctacccactg ggaagtatgg

7651 ctgctgtcca atgcccaatg ccatctgctg ttccgaccac ctgcactgct

7701 gcccccagga cactgtatgt gacctgatcc agagtaagtg cctatccaag

7751 aactacacca cggatctcct gaccaagctg cctggatacc cagtgaagga

7801 ggtgaagtgc gacatggagg tgagctgccc tgaaggatat acctgctgcc

7851 gcctcaacac tggggcctgg ggctgctgtc catttgccaa ggccgtgtgt

7901 tgtgaggatc acattcattg ctgcccggca gggtttcagt gtcacacaga

7951 gaaaggaacc tgcgaaatgg gtatcctcca agtaccctgg atgaagaagg

8001 tcatagcccc cctccgcctg ccagacccac agatcttgaa gagtgataca

8051 ccttgtgatg acttcactag gtgtcctaca aacaatacct gctgcaaact

8101 caattctggg gactggggct gctgtcccat cccagaggct gtctgctgct

8151 cagacaacca gcattgctgc cctcagggct tcacatgtct ggctcagggg

8201 tactgtcaga agggagacac aatggtggct ggcctggaga agatacctgc

8251 ccgccagaca accccgctcc aaattggaga tatcggttgt gaccagcata

8301 ccagctgccc agtagggcaa acctgctgcc caagcctcaa gggaagttgg

8351 gcctgctgcc agctgcccca tgctgtgtgc tgtgaggacc ggcagcactg

8401 ttgcccggcc gggtacacct gcaatgtgaa ggcgaggacc tgtgagaagg

8451 atgtcgattt tatccagcct cccgtgctcc tgaccctcgg ccctaaggtt

8501 gggaatgtgg agtgtggaga agggcatttc tgccatgata accagacctg

8551 ttgtaaagac agtgcaggag tctgggcctg ctgtccctac ctaaagggtg

8601 tctgctgtag agatggacgt cactgttgcc ccggtggctt ccactgttca-

8651 gccaggggaa ccaagtgttt gcgaaagaag attcctcgct gggacatgtt

8701 tttgagggat ccggtcccaa gaccgctact gtag
